# COVID-19—Zoonosis or Emerging Infectious Disease?

**DOI:** 10.3389/fpubh.2020.596944

**Published:** 2020-11-26

**Authors:** Najmul Haider, Peregrine Rothman-Ostrow, Abdinasir Yusuf Osman, Liã Bárbara Arruda, Laura Macfarlane-Berry, Linzy Elton, Margaret J. Thomason, Dorothy Yeboah-Manu, Rashid Ansumana, Nathan Kapata, Leonard Mboera, Jonathan Rushton, Timothy D. McHugh, David L. Heymann, Alimuddin Zumla, Richard A. Kock

**Affiliations:** ^1^The Royal Veterinary College, University of London, Hertfordshire, United Kingdom; ^2^Department of Epidemiology and Population Health, Institute of Infection, Veterinary and Ecological Sciences, University of Liverpool, Liverpool, United Kingdom; ^3^Division of Infection and Immunity, Department of Infection, Centre for Clinical Microbiology, University College London, London, United Kingdom; ^4^Epidemiology and One Health, Animal Health Policy Branch, Department of Agriculture, Water and the Environment, Canberra, ACT, Australia; ^5^Bacteriology Department, Noguchi Memorial Institute for Medical Research, University of Ghana, Accra, Ghana; ^6^Department of Community Medicine, Njala University, Bo, Sierra Leone; ^7^Zambia National Public Health Institute, Ministry of Health, Lusaka, Zambia; ^8^South African Centre for Infectious Diseases Surveillance (SACIDS) Foundation for One Health, Sokoine University of Agriculture, Morogoro, Tanzania; ^9^Infectious Disease Epidemiology, London School of Hygiene & Tropical Medicine, London, United Kingdom; ^10^National Institute of Health Research, Biomedical Research Centre, University College London Hospitals, National Health Service Foundation Trust, London, United Kingdom

**Keywords:** COVID-19, SARS-CoV-2, zoonoses, emerging infectious disease (EID), spillover

## Abstract

The World Health Organization defines a zoonosis as any infection naturally transmissible from vertebrate animals to humans. The pandemic of Coronavirus disease (COVID-19) caused by SARS-CoV-2 has been classified as a zoonotic disease, however, no animal reservoir has yet been found, so this classification is premature. We propose that COVID-19 should instead be classified an “*emerging infectious disease (EID) of probable animal origin*.” To explore if COVID-19 infection fits our proposed re-categorization vs. the contemporary definitions of zoonoses, we reviewed current evidence of infection origin and transmission routes of SARS-CoV-2 virus and described this in the context of known zoonoses, EIDs and “spill-over” events. Although the initial one hundred COVID-19 patients were presumably exposed to the virus at a seafood Market in China, and despite the fact that 33 of 585 swab samples collected from surfaces and cages in the market tested positive for SARS-CoV-2, no virus was isolated directly from animals and no animal reservoir was detected. Elsewhere, SARS-CoV-2 has been detected in animals including domesticated cats, dogs, and ferrets, as well as captive-managed mink, lions, tigers, deer, and mice confirming zooanthroponosis. Other than circumstantial evidence of zoonotic cases in mink farms in the Netherlands, no cases of natural transmission from wild or domesticated animals have been confirmed. More than 40 million human COVID-19 infections reported appear to be exclusively through human-human transmission. SARS-CoV-2 virus and COVID-19 do not meet the WHO definition of zoonoses. We suggest SARS-CoV-2 should be re-classified as an EID of probable animal origin.

## Introduction

The phenomenon of “*spill-over”* or “*evolutionary jump*” refers to the transmission of a pathogen from a natural animal host to a novel host leading to infection in the new host. This may transpire by chance, novel exposure, repeated exposure, or key genomic change enabling the pathogen to infect the new host (1). Infection in the new host can result in a dead-end or can lead to spread through secondary epidemiological cycling to conspecifics, or even zooanthroponotic transmission as is the case with COVID-19. Spill-over is a chance event rather than a normal part of organism infection cycles. In popular terminology cross-species spill-over, where it becomes established, is defined as a pathogen jump from animals to humans (1). Spill-over is illustrated by human immunodeficiency virus (HIV) and Ebola in recent decades, and yellow fever, dengue, measles, and smallpox in the past centuries (2).

The term zoonosis is very plainly defined by the World Health Organization (WHO) as “*any infection that is naturally transmissible from vertebrate animals to humans*” (3). This is qualified by stating that the infection is maintained in an animal population (a reservoir) and therefore a continuous source of human infection (3). This encompasses infections that are acquired by humans through direct contact with animals, as well as infections transmitted through indirect exposure routes such as vector-borne or environmental and food system pathogens. An example of a zoonosis is rabies, which is almost entirely transmitted by the bite of an infected dog with exceptionally rare spill-over from wild animals (4). Wildlife are defined by the World Organization for Animal Health (OIE) as any of the followings: (a) wild animals (phylogenetically distinct wild animal species, free-ranging), (b) feral [domesticated] animals, free-ranging, and (c) non-domestic animals in captivity or farming. However, although the WHO definition does delineate between diseases that originate in animals but independently persist in human populations, vs. diseases that require a non-human animal host for pathogen survival and persistence, there is no term to describe the former and contemporary literature often incorrectly terms the former as well as the latter diseases as zoonoses.

Emerging infectious diseases (EIDs) are currently defined as “*Diseases that have newly appeared in a population or have existed but are rapidly increasing in incidence or geographic range*” (5) with the period of emergence appearing somewhat open for interpretation. This definition does not distinguish between different categories of emergence or re-emergence and thus does not reflect the very different drivers and significance between diseases and pathogens in terms of global burden, threat and origin. Further, it does not necessarily differentiate the relatively uncommon but concerning new diseases with pandemic impact or potential such as SARS, COVID-19 or MERS-CoV from those which are simply variants of old pathogens, new detections of old pathogens with new tools, or re-emergence of old pathogens in new geographies, acknowledging that these may be locally important. Thus, the term EID as it stands is unhelpful at best, and at worst easily misinterpreted. Although the ambiguity undoubtedly needs to be addressed at greater scale, we suggest an interim solution for the classification of COVID-19 is to designate it an “*EID of probable animal origin*.” This acknowledges its status as an emerging human pathogen while allowing for the possibility that it spilled-over from an animal reservoir but stops short of misrepresenting it as a zoonotic disease. EIDs are not necessarily of animal origin, however categorizing them correctly allows it for distinction and ultimate focus and allocation of resource attention. Most diseases classified as EIDs over the last few decades [e.g., (6)] are variants of known pathogens, new detections of pathogens with new technologies, or known pathogens which have emerged or re-emerged in new geographies. Examples include: *Enterococcus faecalis* var-Gentamycin resistant, *Escherichia coli* O157:H7 (novel variants), West Nile virus, and Zika virus (changing geographies).

Differentiating between diseases that may originate in animals but independently persist in human populations, vs. diseases that require a non-human animal host for pathogen survival and persistence will enable more targeted and strategic initiatives in infectious disease research, policy, prevention, and control. Additionally, greater specificity and distinction between these types of pathogens will avoid the confusion and misrepresentation that arises from classifying the majority of relatively low-impact, rare zoonotic infections that spill-over from wild animals under the same zoonotic designation as the more common [ongoing] zoonotic transmissions from domestic animals and captive wildlife species. This will clarify the common narrative that ~60% of emerging infectious diseases are of zoonotic origin and ~70% of these originate from wildlife (which includes all wild animals, feral animals and captive or farmed wildlife—as defined earlier) (6), when in fact only very few diseases of wild animal origin persist with ongoing zoonotic transmission. The World Health Organization (2020) currently list the EIDs of epidemic concern in their research and development blueprint as COVID-19, Crimean Congo haemorrhagic fever (CCHF), Ebola and Marburg viruses, Lassa Fever, MERS, and SARS coronaviruses, Nipah and henipaviral diseases, Rift Valley fever (RVF), Zika and “Disease X” (“*a serious international epidemic could be caused by a pathogen currently unknown to cause human disease”*) (7). Of these, the only ongoing zoonoses are extremely rare sylvatic spill-over cases of Marburg, CCHF and henipaviruses from wild animals, and RVF and MERS cases from livestock. Lassa Fever is acquired from a peri-domestic rodent, while the source of Ebola virus, SARS, and SARS-CoV-2 remain enigmatic with the vast majority of human infections from these diseases acquired through human-human transmission.

The pandemic of Coronavirus disease 2019 (COVID-19) caused by SARS-CoV-2 has been designated a zoonotic disease (8, 9). The SARS-CoV-1 emergence in 2002 was similarly defined as a zoonotic disease, however, despite identification of over 500 beta (β) coronaviruses from bats in the region of emergence and surrounding area, no reservoir has been definitively confirmed (6). This paper argues for a correction of the current biased narrative around zoonoses through an examination of the COVID-19 pandemic and further demonstrates that, unlike conventional zoonoses which can be relatively intractable (e.g., rabies), EID emergence has been consistently linked to human pressures on ecosystems largely through our food systems (especially livestock), suggesting that EIDs may be preventable (6, 10). This article further explores SARS-CoV-2 transmission evidence across species and suggests that COVID-19 should instead be classified as an “*emerging infectious disease of probable animal origin*.”

## Methods

**Figure 1:**
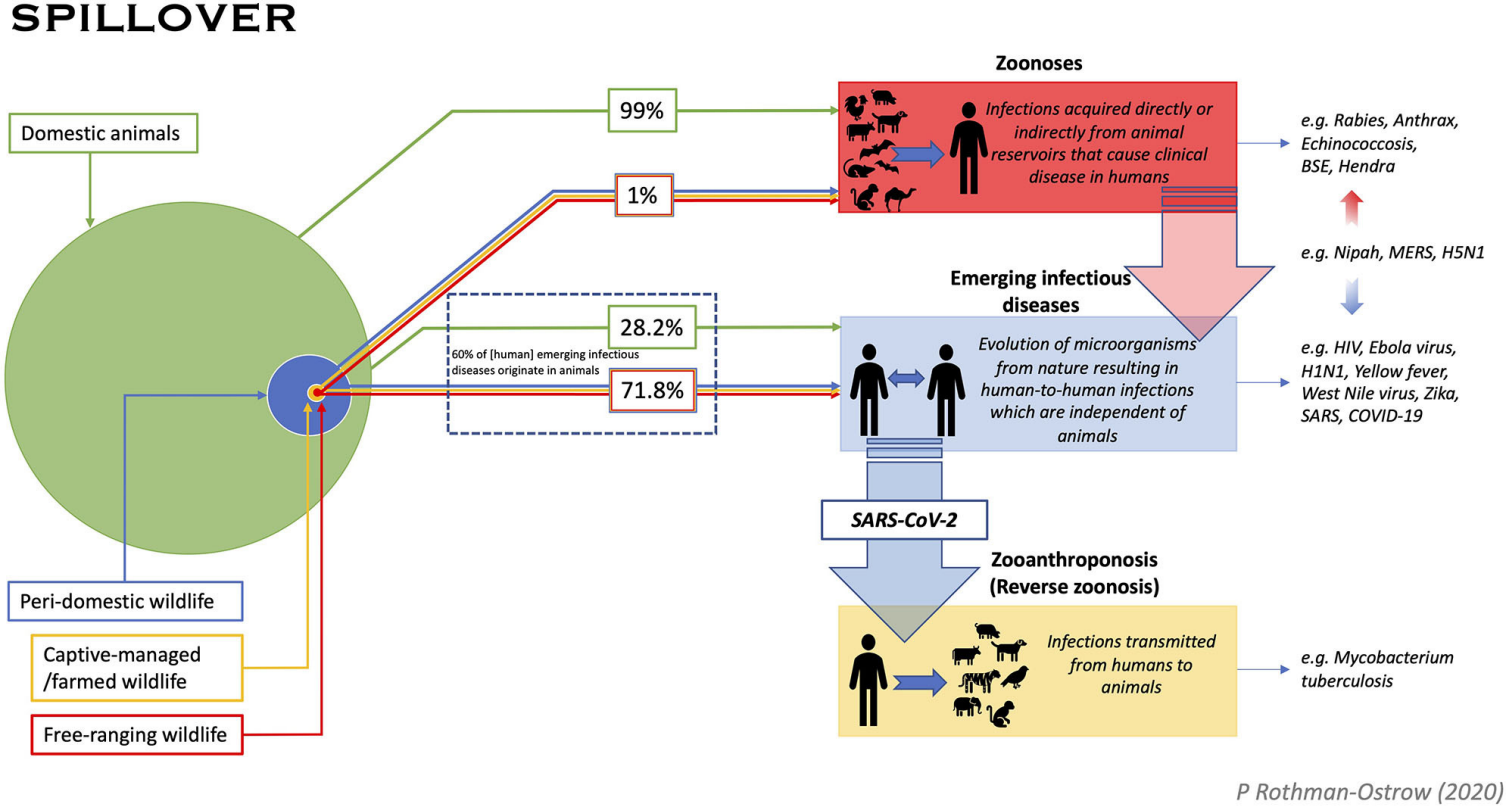
Pathway diagram for pathogen spill-over to humans from animals describes three distinct processes. (1) Zoonoses: Pathogens that are transmitted from an animal reservoir directly or indirectly (e.g., foodborne, vector-borne, etc.) to humans causing disease; (2) Emerging Infectious Diseases: Pathogens that cause an emergent infectious disease in humans and persist in human populations irrespective of an animal reservoir. Genetic origins may show links to non-human animals, but these diseases undergo a more complex process of evolution not necessarily dependent on a specific animal reservoir, and usually evolve to be independent of animals, (3) Zooanthroponosis: “Reverse zoonosis” whereby humans transmit infection to animals. Infected animals, may or may not then go on to circulate the pathogen or establish a disease within conspecific population. A disease can fall into more than one category as exemplified in the figure by Nipah, MERS, and SARS-CoV-2. Spill-over origin for zoonoses and emerging pathogens (i.e., animals) is given proportion by the size of the circles. The relative infection frequency of spill-over from domestic animals and wildlife is quantified in the percentages shown. Proportion of animals was determined from a review of the literature which found that only 4% of global mammalian biomass is wild, of which >50% is estimated to be marine mammals, with livestock making up ~60% of the remainder (39). Percent of emerging pathogens coming from wildlife was derived from the assessment that ~60% of EIDs are zoonotic, with 71.8% of those found to have wildlife genetic origins (6). Percent zoonoses derived from domestic vs. wild species is a rough estimate based on available data from a variety of publications and considers the following: zoonoses have been estimated to cause 2.5 billion cases of human illness, 2.4 billion of which are caused by thirteen diseases, all of which bear relevance to agriculture (40); zoonotic viral richness is strongly correlated with mammalian species diversity and abundance with domestic species found to harbor, on average, 19.3 zoonotic viruses compared to an average of 0.23 harbored by wildlife, suggesting wildlife harbor >0.5% of viral diversity (43). In addition, an analysis of mammalian species with the greatest number of viruses shared with humans found the top eight species to be domestic (43). Our estimate that 99% of zoonotic diseases spill over from domestic animals is considered reliable for direct zoonotic transmission and indirect foodborne infection, though less reliable for vector borne diseases.

To explore if the COVID-19 infection fits our proposed categorization as an EID of probable animal origin, we reviewed current evidence and the designation of different diseases as zoonoses and EIDs as well as the classification of simple spill-over events. We also sought to assign origin percentages to pathogen emergence in consideration of domesticated animals vs. wildlife with the latter separated into peri-domestic, captive-managed/farmed, and free-ranging groupings (see earlier OIE definition of wildlife) (Figure 1). Domesticated animals are defined as those used for food production, draft power, sport, or companion animals. Peri-domestic wildlife are defined as animals adapting to human landscapes and living in close proximity to people including animals brought into human habitation as food. Captive-managed/farmed non-domestic and peri-domestic wildlife species are mostly from the rodent, primate, carnivore, herbivore, bird and bat taxa and are the most important from the perspective of zoonoses and EIDs.

## Results and Discussion

The COVID-19 pandemic is an infection caused by SARS-CoV-2, a novel coronavirus first detected in Wuhan city, Hubei province, China at the Huanan Seafood Wholesale [Wet] Market where epidemiological investigation found that approximately one hundred of the initial patients were exposed (11–14). Animal food commodities from over one hundred domestic and wild species such as bats, mink, fox, wolves, snakes, Chinese bamboo rats, civet cats, raccoon dogs, cats, porcupines, dogs, poultry, camels, and other farm animals as well as fish were reportedly sold at this location and at hundreds of similar markets across the region. Live purchase and slaughter also occurred on all premises (11).

Evidence for the market being a point source outbreak of SARS-CoV-2 is based on the tracing of patients' movements and reports that 33 of 585 swab samples collected from surfaces and cages in the market tested positive for SARS-CoV-2 (15). The majority of positive samples (*n* = 31) were taken from stalls that traded animals designated as “wildlife,” although most of the animals may have been reared in captivity (15). Regrettably, no live animal or animal product sampling for SARS-CoV-2 at the market has been reported. Chinese authorities subsequently implemented a national ban on wildlife trade with exceptions for fur, medicine, and research (16). This was done before forensic examination of the wildlife industry for SARS-CoV-2 was possible. As of mid-October 2020, it is likely that SARS-CoV-2 initially spilled-over directly or indirectly from animals or animal-based food products to humans in China via the Wuhan market or potentially a similar source such as restaurants or home deliveries (17), farms, and/or other wildlife product supply chains. However, the initial spill-over event cannot now be easily proven and human origin virus brought into the market may also be a source of point epidemics as is believed to be the case in later resurgences such as at a market in Beijing (18), meat factories and similar industries. Serological surveillance of bat workers and guano farmers in southern China showed a low-level of seroconversion to SARS-like coronavirus-specific antibodies (19), suggesting that direct transmission from bats is also possible, though no ill health was associated with patient exposure. In contrast, SARS research in 2003–2004 showed animal food trade workers to be infected or possess anti-SARS antibodies, suggesting SARS virus spill-over to workers from confirmed-infected animal species and or zooanthroponosis (reverse zoonosis) (20). In South East Asia, wildlife supply chains for human consumption and other products have been associated with increased disease transmission risk but without proven zoonosis cases (21). Some 14 million Chinese work in the wildlife farming industry which encompasses the fur trade and was valued at approximately $73 billion USD by a Chinese Academy of Engineering report in 2017. This collective industry may constitute by far the greatest infection risk from all wildlife in the region (22).

Phylogenetic analysis of the β-coronavirus genera indicated that SARS-CoV-2 is similar to some viruses identified in bats in a group described as SARS/SARS-like CoV (23). The SARS-CoV-2 virus is 96% identical at the whole-genome level to a coronavirus isolated from horseshoe bats (*Rhinolophus affinis*) in Yunnan province, China (23) and its evolution suggests common ancestry approximately 50 years ago (24). A coronavirus identified in a Malayan pangolin (*Manis javanica*) was also found to share a 91% identical sequence at full genome level to SARS-CoV-2 (25). Further bioinformatic analysis allowes the identification of a unique peptide insertion (PRRA) in the spike protein of SARS-CoV-2, however this insertion is absent in the spike protein of the coronavirus identified in pangolins (termed Pangolin CoV) (26). Throat and rectal swabs collected from 334 Sunda pangolins (Manis javanica) in Malaysia between 2009 and 2019 were all negative for *Coronaviridae* (27), suggesting that pangolins might have been contaminated with coronavirus in the wildlife trade network (27). Thus, although they are widely traded throughout South and South East Asia, scientists concluded that SARS-CoV-2 was unlikely to have spilled-over directly from pangolins (28).

At present, the possible animal source of SARS CoV-2, or the transitional virus it may have evolved from, remains unknown. Genetic features indicate that SARS-CoV-2 could have resulted from natural selection in animal species before its evolutionary jump into humans, or that after a zoonotic transfer the virus was naturally selected within the human population. However, there is a dearth of information to indicate which hypothesis reflects the eventual outcome (26) and no conclusive evidence for any of these routes has so far been found. Therefore, it is reasonable to assume that SARS-CoV-2 has an animal origin in an evolutionary sense (26), but there is no categorical proof to suggest it should be characterized as a directly- or indirectly-transmitted zoonotic disease.

In the months after COVID-19 was reported on all continents (except Antarctica) and designated a human pandemic by the WHO, SARS-CoV-2 has been detected in a small number of animals who had been exposed to infected humans or challenged experimentally. These include domesticated cats, dogs, and ferrets, and captive-managed mink, lions, and tigers, suggesting zooanthroponosis (8, 29–32). Four domesticated cats (one each in Hong Kong, Belgian, French and American cities) and three dogs (two in Hong Kong, and one in the USA) all belonging to COVID-19-positive owners tested positive (29). Experimental studies have shown that cats, ferrets, and primates are susceptible to infection with SARS-CoV-2 and can transmit the virus. Zoo animals including a tiger and a lion were reported to have been infected by their zoo career (8, 33, 34). Zooanthroponotic infections (human to mink) occurred in the Netherlands, Denmark and Spain, and mink-to-human transmission has been suggested (29, 35) leading to the slaughter of all mink on infected farms. This raises the possibility of wider dissemination and future involvement of multiple species in SARS-CoV-2 circulation and persistence, but few studies have been completed thus far (36–38). Thus, evidence to date indicates that SARS-CoV-2 should not be considered a zoonosis and wildlife should not be the default culprit not only for COVID-19 but for other inappropriately labeled zoonoses.

The definition of zoonoses is important in the story of SARS-CoV-2, as a precise understanding of its origins and epidemiology is vital to determining the risk of spill-over recurring and the drivers of such an event. Furthermore, this precise understanding is also important for determining the risk of spill-over and drivers for other pathogens of pandemic potential. In order to inform policy and target research it is important to differentiate spill-over from domesticated animals vs. from wildlife and in turn differentiate free-roaming wildlife from captive-managed animals that may have wild origins but have either been caught and maintained or bred in captivity.

A distinction between EIDs and zoonotic diseases in terms of spill-over origin from domesticated vs. wildlife biomass is represented in Figure 1. The proportion of domestic to wild animals was determined from a review of the literature which suggests that only 4% of global mammalian biomass is wild, of which >50% were estimated to be marine mammals (39) with livestock making up ~60% of the remainder (39). Humans comprise the remaining 36% of mammalian biomass, but in considering non-human mammals alone, these estimates suggest that wildlife make up <1% of the world's non-human mammal biomass. It is also worth considering that these ratios may differ greatly between different geographies and also temporally as populations fluctuate with breeding seasons. Nonetheless, these figures augmented the estimates for percent zoonoses derived from domestic vs. wild species, which is a rough estimate that considers the following additional points: zoonoses have been estimated to cause 2.5 billion cases of human illness, 2.4 billion of which are caused by thirteen diseases of which nine have high impact on livestock (40). Zoonotic diseases most frequently cited as “high impact” are endemic zoonoses such as rabies, brucellosis and cysticercosis which have clear proven transmission links with domestic animals (40). For instance, of the estimated 59,000 annually-reported human rabies cases, 99% are transmitted through the bite of an infected dog (41). While it's been estimated that 88.6% of terrestrial mammals have yet to be diagnosed with a zoonotic virus, zoonotic viral richness is strongly correlated with mammalian species diversity and abundance (42). For instance, 50% of zoonotic virus richness detected thus far has been found in domesticated species with these animals hosting an average of 19.3 viral species each, compared to wild species surveyed harboring an average of 0.23 (42). This suggests that wildlife harbor >0.5% of viral diversity (42). The study by Johnson et al. (43) also associated increased viral abundance with growing domesticated species population in proximity to humans, namely changes in livestock food systems in response to increased urban-based demand for animal products (43). While it is acknowledged that this is an educated assumption, we argue that our estimate that 99% of zoonotic diseases spill-over from domesticated animals, is reliable for direct zoonotic transmission and indirect foodborne infection, though less reliable for vector borne diseases.

In considering EIDs, a 2008 study by Jones et al., reviewed EID origin from 1940 to 2004 and found that 60% of EIDs originate in animals (6). Of the animal-origin EIDs, 71.8% were found to have wild animal genetic origins, suggesting that 28.2% to jump to humans from domesticated species [(6); Figure 1]. As illustrated in Figure 1, we also explored the term wildlife in the context of peri-domestic, captive/managed farmed animals and free-roaming.

In considering the proposition that bush meat poses zoonotic transmission risk (42), much of the wildlife [game] industry in the southern Africa should not be considered free-ranging wildlife, nor bush meat by definition, but rather wild animal farming. It is likely this industry is a source of general zoonotic pathogens that are seen in conventional livestock food systems given that they often live side by side and share similar husbandry and management risks in terms of intensification (44). Similarly, in the case of most “wild” animals traded in markets, it is important to distinguish that they were not from wild-living populations, but from farmed or captive populations of species that are not fully domesticated but are kept in domestic conditions (11). The pathogenesis of infections in these densely populated farms and live markets such as those harboring civet cats and raccoon dogs or mink in the meat and fur trade, will be very different to infection cycles in natural [wild] populations. These crowded environments, similar to intensive [livestock] farming, are likely incubators of spill-over-disposed pathogens such as SARS-like viruses (45). Bats most likely have a role in this story as originators of progenitor viruses, but it is likely a result of their close contact with other species through wildlife trade or directly with humans that facilitated dissemination.

The SARS-CoV-1 event of 2002–2004 and the sudden emergence of SARS-CoV-2 from a similar virus leading to its subsequent pandemic spread in humans suggests these events are likely end points in an ongoing development process (46). Therefore, it can be expected that without changes to the pathogen evolutionary landscape, similar future events are inevitable. In the quest to prevent this occurrence, research should focus on understanding the wider environmental and societal structures that foster emergence of novel diseases. In prioritizing areas for immediate attention, we propose launching an ongoing investigation into the animal-based food systems (including wildlife) in an effort to identify: (1) Zoonotic disease (as defined by the WHO) risks; (2) Investigate potential intermediate amplifying hosts from known viral reservoirs; (3) Implement the International Union for Conservation of Nature (IUCN) and OIE (47) risk analysis methodologies for pathogen emergence and zoonosis from the wildlife trade industry, including wider analysis of anthropogenic drivers to identify vital factors which can be addressed expediently; (4) Encourage social distancing between wildlife and humans (mutually beneficial); and (5) Apply international domesticated animal food zoosanitary standards on all species used for food, globally. As an early attempt on how this can be achieved there is a need to combine risk analysis with value chain analysis (48).

A key thread linking these prioritizes, is the direct relationship between the economy, disease and how we react to the presence or risk of a disease, a fact that has been underscored by the COVID-19 pandemic. Understanding this relationship in the context of zoonotic diseases is challenging and requires the evaluation of the economic impacts of disease on domesticated species such as livestock, alongside the human health impacts (49). Livestock production is an economic activity with positive and negative public health implications where disease impacts can be monetarised, whereas human health loss has challenges with a reluctance to place a monetary value on ill health particularly given that cost of treatment and loss of wages can vary depending on geographic and demographic indicators (49).

Human health has adopted the Disability Adjusted Life Year (DALY) as a non-monetary measure of illness burden. DALYs are the metric that have been accepted by the WHO as a means of assessing the global burden of human disease, and there is an emerging system for assessing the burden of animal diseases (50). Bringing these approaches together will enhance our capacity for assessing zoonotic disease burden in its human-centric approach, but reduces efficacy of disease burden estimations and health costs in the animal sector (51, 52).

Irrespective of the lack of synergy in economic impact tools across species, it is undisputed that economics directly influence pathogen emergence and spread through trade, tourism, urbanization and globalization (53, 54). In turn, epidemics and pandemics influence local, national and global economies (55). Once a pathogen has emerged as an epidemic or pandemic threat, a clear understanding of the events that spawned outbreak and persistence in terms of pathogen evolution, transmission routes and epidemiology is vital for evaluation of economic impact and related policy development.

The classification of COVID-19 as a zoonotic disease, and the fact that its emergence was linked to the Huanan [wet] Seafood Market, led China to shut down and ban the farming and sale of wildlife for human consumption. This has been lauded by many and may well have been the correct tactic as a precautionary measure, but the impacts on economy and livelihoods may be unjustified until the risk is more comprehensively understood. Indeed, coupled with erasing forensic evidence for evaluation of SARS-CoV-2 emergence, severe economic implications may follow, particularly for lower income communities who have been encouraged in recent years by government agencies to invest family savings into initiating wildlife farming (56, 57). It is also likely that a ban on wildlife food trade will strengthen that black market industry (56, 57). These probable consequences will come with their own set of ramifications and may not address the underlying problems of disease emergence when we explore SARS-CoV-2 as an “*EID of probable animal origin*”.

The terms zoonosis and EID are not necessarily mutually exclusive. For example, MERS virus meets the criteria for a zoonosis with the Dromedary camel serving as the known animal reservoir and natural animal-human transmission recorded consistently in Middle Eastern countries since 2012. MERS also fits the definition of EID as it can be transmitted between humans and emerged as an infectious disease with no evidence of historical infections. Similarly, EIDs may be of human not animal origin [e.g., Hepatitis B virus (2)].

## Conclusion

Current narratives around wildlife and zoonoses are biased and disproportionate. However, increasing evidence for novel emerging pathogens of humans beyond an historic norm requires forensic examination of their origin and epidemiology. At the time of writing (mid-October, 2020), more than 40 million confirmed human cases of COVID-19 have been reported to the WHO (58). It is likely that only a few direct or indirect zoonotic transmissions of SARS-CoV-2 virus occurred from unknown animals or their products. It is hypothesized that such transmission led to a point source outbreak at the Huanan Seafood Market in China resulting in wider human-human transmission, which caused the pandemic. Since the transmission—directly or indirectly—of the virus between animals and humans, and a reservoir—if one exists—is unknown, we argue that strictly speaking, it should not be termed a zoonosis, but rather COVID-19 should be classified an “*EID of probable animal origin.”* It is evident the virus possesses the ability to transmit between humans without requiring maintenance in a separate reservoir species and can transmit zooanthroponotically. Re-classification of COVID-19 as an EID makes it no less valuable or imperative that research confirm whether an animal reservoir actually exists especially considering that if one does, it could become a potential source of future human infection. Moreover, it will influence not only the ongoing research and response to COVID-19 specifically, but will reshape and revamp the way the international community addresses future pandemic preparedness and threats. Additionally, withdrawing the ill-suited designation of SARS-CoV-2 as a zoonosis will reduce the risk of inappropriate animal persecution or other unsuitable interventions whilst the source of the problem or animal associated risk is unknown. In their emergence, EIDs have historically been known to share several key characteristics regardless of pathogen type: high morbidity, explosive growth and spread, and grave social impacts (53). We therefore argue that it is imperative to review COVID-19 predominantly as an EID in order to address the underlying drivers of the emergence of such pathogens which can be so readily driven, yet so easily adjusted by human activities. In closing, we propose that a whole of society debate around the designation of a disease as a zoonosis vs. an EID as well as the nuances within each term is needed to reduce the risks of disease events such as COVID-19 in the future through appropriate actions in the human political landscape.

## Data Availability Statement

The original contributions presented in the study are included in the article/supplementary materials, further inquiries can be directed to the corresponding author/s.

## Author Contributions

NH and RK: conceptualization and preparing the first draft manuscript. NH, RK, and PR-O: methodology and literature review. PR-O, RK, AYO, LBA, LE, MJT, DY-M, RA, NK, LM, JR, TDM, DLH, AZ, and LM-B: writing review and editing. NH, PR-O, RK, and LM-B: address the comments of reviewers and editors. All authors contributed to the article and approved the submitted version.

## Conflict of Interest

The authors declare that the research was conducted in the absence of any commercial or financial relationships that could be construed as a potential conflict of interest.
